# The Role of Extracellular Vesicles: An Epigenetic View of the Cancer Microenvironment

**DOI:** 10.1155/2015/649161

**Published:** 2015-10-25

**Authors:** Zhongrun Qian, Qi Shen, Xi Yang, Yongming Qiu, Wenbin Zhang

**Affiliations:** ^1^Department of Neurosurgery, Ren Ji Hospital, School of Medicine, Shanghai Jiao Tong University, Shanghai, China; ^2^Department of Medical Oncology, Sir Run Run Shaw Hospital, School of Medicine, Zhejiang University, Hangzhou, Zhejiang, China; ^3^Department of Neurosurgery, Nanjing Brain Hospital, Nanjing Medical University, No. 264, Guangzhou Road, Nanjing, Jiangsu 210000, China

## Abstract

Exosomes, microvesicles, and other extracellular vesicles are released by many cell types, including cancer cells and cancer-related immune cells. Extracellular vesicles can directly or indirectly facilitate the transfer of bioinformation to recipient cells or to the extracellular environment. In cancer, exosomes have been implicated in tumor initiation, proliferation, and metastasis. Extracellular vesicles can transmit proteins and nucleic acids that participate in DNA methylation, histone modification, and posttranscriptional regulation of RNA. Factors transmitted by extracellular vesicles reflect the donor cell status, and extracellular vesicles derived from tumor cells may be also responsible for altering expression of tumor promoting and tumor suppressing genes in recipient cells. Thus, circulating extracellular vesicles may act as biomarkers of cancer, and detection of these biomarkers may be applied to diagnosis or assessment of prognosis in patients with cancer.

## 1. Introduction

Extracellular vesicles include a variety of nanoscale membranous vesicles [1] released by many cell types into the intercellular microenvironment [2, 3]. Subtle changes in the cellular microenvironment may stimulate malignant transformation of cells, and cellular microenvironment has been implicated in tumor initiation, proliferation, and metastasis. Transformed cancer cells may disseminate bioinformation in autocrine and paracrine manners, to help the cancer proliferate and metastasize. This cell-cell or cell-microenvironment communication may be achieved by direct contact or over longer distances by secreted molecules and secreted membranous vesicles. A growing body of evidence suggests that cancer cells release more extracellular vesicles than healthy cells [4–6], partly due to activation of certain oncogenes, including* ras* [7].

Whether individual extracellular vesicles participate in normal physiological regulation or promotion of pathological processes is dependent on what they contain [8–10]. Exosomes, microvesicles and other extracellular vesicles differ in properties such as size, morphology, buoyant density, and protein composition [11]. Exosomes range in size from 40 to 1000 nm, and microvesicles are >1000 nm. Microvesicles bud directly from the plasma membrane, whereas exosomes are derived from endosomes and are released from cells by fusion of the multivesicular endosome with the plasma membrane [2]. Due to their endosome origin, exosomes contain endosome-associated proteins. Exosomes, microvesicles, and other extracellular vesicles can contain proteins, RNA, DNA, and lipids [12], and thus can deliver these factors to the intercellular environment or recipient cells. Extracellular vesicles are transported in the blood, urine, ascites, and cerebrospinal fluid [13–16] and thus may deliver their contents to either neighboring or distant recipient cells and produce corresponding physiological or pathologic effects. For example, melanoma-derived exosomes can deliver the receptor tyrosine kinase MET oncoprotein to bone marrow progenitor cells, which directs their development toward a prometastatic phenotype [17].

The content of extracellular vesicles, therefore, may be clinically relevant to disease progression, and detection of extracellular vesicles may be useful in diagnosis of cancer and assessment of prognosis. Circulating microvesicles and exosomes have been detected in the blood samples of patients with glioblastoma [18], colorectal cancer [19], and ovarian cancer [20]. These microvesicles and exosomes contain bioinformation reflecting primary tumor mutations and can act as early indicators of drug efficacy.

Epigenetic regulation involves processes causing functionally relevant changes to the genome which do not alter the nucleotide sequence but do alter gene expression. The genome plays a significant role in the tumor microenvironment, and the microenvironment influences cancer initiation, proliferation, and metastasis [21]. Important mechanisms of epigenetic regulation such as DNA methylation, histone modification and microRNA (miRNA), or long noncoding RNA (lncRNA) regulation are hot topics in cancer research. Recent research has implicated extracellular vesicles in epigenetic regulation of cancer progression [22, 23]. Gene ontology (GO) analysis has indicated that many mRNAs and proteins contained in extracellular vesicles are involved in epigenetic regulation [24], and exosome-mediated transfer of miRNAs is considered to be an important mechanism of genetic exchange between cells [25]. Thus extracellular vesicles regulate epigenetic processes including DNA methylation, histone modification, and miRNA or lncRNA regulation, and the resultant epigenetic modifications are responsible for changes in the expression of tumor promoting genes and tumor suppressing genes. Recent research indicated that the detection of epigenetic biomarkers, such as miRNAs, in extracellular vesicles could be exploited for diagnosis of cancer or assessment of cancer prognosis [26]. In this review we discuss the role of extracellular vesicles in transmission of factors responsible for three forms of epigenetic regulation: DNA, histone, and noncoding RNA modification.

## 2. The Role of Extracellular Vesicles in DNA Methylation and Demethylation in the Cancer Microenvironment

Epigenetic DNA modification of oncogenes or antioncogenes is crucially important for the initiation, proliferation, and metastasis of many tumors [27–29]. Dynamic variation in DNA methylation is one of the most universal factors influencing transcription of oncogenes and antioncogenes. DNA methyltransferase 1 (DNMT1), DNA methyltransferase 3a (DNMT3a), and DNA methyltransferase 3b (DNMT3b) add methyl groups to specific cytosines in the CpG islands of regulatory sequences, thus silencing certain genes [30]. The level of oncogene or antioncogene transcription fluctuates according to promoter methylation status, thus dynamically affecting tumor progression. Enzymes that demethylate DNA, such as activation-induced cytidine deaminase (AICDA) [31] and thymine DNA glycosylase (TDG) [32], protect unmethylated regions of mammalian genomes from* de novo* methylation.

Due to their complex bioactive cargo, extracellular vesicles can cause malignant transformation of normal cells. The protein, DNA, or RNA contained in extracellular vesicles could induce epigenetic changes in recipient cells by affecting the methylation status of their genome. Microvesicles are extracellular vesicles of 100–1000 nm in diameter [2, 33] which bud from the plasma membrane of tumor cells, while exosomes are 30–100 nm in diameter and are derived from the endosome [2, 34]. Microvesicles released from leukemia cells have been demonstrated to increase global DNA methylation levels in recipient cells. Hypermethylation of the promoter regions of tumor-suppressor genes* P53* and* RIZ1* was observed in cells incubated with leukemia-derived microvesicles and attributed to the increased level of DNMT3a and DNMT3b mRNA and protein [34]. Interestingly, protein and mRNA levels of activation-induced cytidine deaminase (AICDA), a deaminase involved in DNA initiative demethylation, was also increased in recipient cells. These results indicate that genomic instability was promoted in recipient cells, which might further induce leukemic transformation [34]. When microvesicles were treated with RNase, the level of DNMT3a, DNMT3b, and AICDA decreased, indicating that leukemia-derived microvesicles influence the methylation status of recipient cells via transmission of microvesicular RNA. Breakpoint cluster region-Abelson leukemia gene human homolog 1 (*BCR-ABL1*) has been reported to be the dominant onco-mRNA in microvesicles released by the K562 leukemia cell line [34]. Extracellular vesicles derived from transformed donor cells may thus transmit enzymes involved in both methylation and demethylation to recipient cells, ultimately inducing alteration in expression of tumor-related genes and accelerating tumor initiation, proliferation, and metastasis.

In addition to inducing malignant transformation of recipient cells, extracellular vesicles may represent useful biomarkers of cancer [35]. Detection of extracellular vesicles, and the molecular markers they contain, in blood or other clinical specimens represents a potentially valuable noninvasive approach for the assessment of tumor initiation, proliferation, and metastasis. Exosomes obtained from the serum of patients with pancreatic cancer were reported to carry genomic double-stranded DNAs (dsDNAs), which contained mutated* KRAS* and* p53* genes [36]. Exosome-associated molecular markers of gastric cancers have been detected in gastric washes and even highly acidic gastric juice [37–39]. Exosomes were purified from the gastric juice of gastric cancer patients, and the content of cancer-related methylated* LINE1* and methylated* SOX17* DNA was analyzed by bisulfite pyrosequencing [37]. The methylation status of* LINE1* and* SOX17* DNA in gastric juice-derived exosomes was found to accurately reflect the methylation status of nuclear DNA in the corresponding tumors, indicating a role in the noninvasive diagnosis of cancer.

## 3. The Role of Extracellular Vesicles in Histone Modification in the Cancer Microenvironment

Chromosomal DNA coils around structural histone proteins to form the basic chromatin structure, which is maintained or altered by histone modification. Relaxation of the chromatin structure will expose more transcriptional regions of chromosomal DNA, inducing gene expression [40, 41]. Histone modification and, frequently, concomitant methylation have been implicated in pathogenic expression of tumor-related genes, and thus chromatic remodeling represents another potentially detectable biomarker for cancer [42]. Posttranslational modification of specific residues in the N-terminal tails of core histones, including acetylation, methylation, ubiquitination, or phosphorylation influence the chromatin shape, and thus transcription of associated genes [43–45]. Methylation of H3K4, H3K48, and H3K79 is commonly associated with gene activation, whereas methylation of H3K9 and H3K27 is associated with gene inactivation [46, 47]. Histone acetylation is regulated by histone acetyltransferase (HAT) and histone deacetylase (HDAC). The latter removes the positive charge on histones, thereby relaxing the condensed chromatin structure to promote gene transcription [48, 49]. The cancer microenvironment may thus influence epigenetic regulation of cancer-related gene expression by DNA modification and histone modification.

The role of extracellular vesicles in histone modification is currently controversial. Bioinformatic analysis has indicated a striking overlap between genes relevant to transgenerational epigenetic inheritance and the contents of exosomes released by a variety of cells, including cancer cells [24, 50–52]. By examining the* GO* biological processes associated with these overlapping mRNAs and proteins, it appears that the exosome content does not affect cellular activities indiscriminately but is focused on a limited network of processes including several processes related to epigenetic modification. Genes relevant to histone acetylation or deacetylation, histone ubiquitination, and other histone modifications, and even chromatin remodeling, represent a remarkably large proportion of those genes determined to be relevant to transgenerational epigenetic inheritance [24]. These findings indicate that exosomal mRNAs and proteins may directly or indirectly participate in both the response to environmental exposure and epigenetic modification, particularly histone modification. Additional analyses of exosomal miRNAs have indicated a similar association between environmental exposure and histone modification [50].

In the cancer microenvironment, these extracellular vesicle-related processes may participate in cancer initiation, proliferation, and metastasis. One cancer cell line, the G26/24 oligodendroglioma cell line, was reported to release extracellular vesicles containing the differentiation-specific linker histone H1°, which is not released by normal astrocytes [53]. The H1 histone family is the most divergent histone family and each H1 protein subtype or variant is associated with specific functions and distributions [54–57]. H1° is mostly associated with terminal differentiation [58, 59]. Although the precise pathophysiological significance of this phenomenon remains to be understood, enrichment of histone H1° in cancer cell-derived extracellular vesicles represents a promising potential molecular marker for oligodendroglioma diagnosis. Furthermore, extracellular vesicles containing histone H1° may subsequently influence recipient cells in the cancer microenvironment.

## 4. The Role of Extracellular Vesicles in Transmitting Noncoding RNA in the Cancer Microenvironment

Noncoding RNA refers to nonprotein coding transcripts, categorized as lncRNAs and small noncoding RNAs, including miRNAs, according to their length. Circulating tumor-associated miRNAs were first detected in the serum of a patient with diffuse large B-cell lymphoma [60], and the potential for circulating noncoding RNAs to act as noninvasive biomarkers of cancer diagnosis spurred accelerated research in this field. Circulating miRNAs were found to be present in plasma in a stable form that was protected from endogenous RNase activity [61], and recently noncoding RNAs were determined to be packaged in extracellular vesicles secreted by tumor cells, explaining why circulating RNAs are stable and thus detectable in the serum [62]. The capacity of extracellular vesicles containing noncoding RNAs to facilitate cell-to-cell communication and alter the cancer microenvironment remains to be seen; however, the potential for noncoding RNAs in extracellular vesicles to reflect the status of cancer cells or cancer-related immune cells is clinically promising. The lncRNAs and miRNAs contained in extracellular vesicles represent promising molecular markers of cancer diagnosis and prognosis assessment.

### 4.1. The Role of Extracellular Vesicles in Transmitting MicroRNAs in the Cancer Microenvironment

MiRNAs are small noncoding RNA molecules of about 22–25 nucleotides which act to silence RNA through posttranscriptional epigenetic regulation [63, 64]. Probably owing to their relatively small size, miRNAs are the most abundant RNA species in exosomes, making up over 42.32% of all raw reads and 76.20% of all mappable reads in 14 size-selected sequencing libraries [65]. The loading of miRNAs into exosomes may be controlled by specific proteins involved in the miRNA network. GW182 is a protein marker of P-bodies which can bind the Argonaute2 (AGO2) protein. The presence of AGO2 protein and striking enrichment of GW182 in purified monocyte-derived exosome-like vesicles suggests the specific and selective loading of miRNAs into exosomes [66–69]. Furthermore the ceramide-dependent machinery has been reported to regulate release of miRNAs [69].

Metastatic cancer cells shed particular types of miRNAs in extracellular vesicles. Microvesicles released from metastatic melanoma cells contain high levels of prominin-1, which promotes metastatic progression [70–72]. Micro-RNA profiling revealed 49 species of miRNA present at higher concentrations in these metastatic-melanoma derived microvesicles than in donor cells, including 20 species of cancer-related miRNAs. The invasiveness of bone marrow-derived stromal cells was found to be increased following exposure to prominin-1 expressing exosomes [73]. In metastatic gastric cancer, the* let-7* miRNA family is selectively secreted into the extracellular environment via exosomes [74], inducing a prometastatic phenotype in selected host tissues. The exosomes released by the metastatic rat adenocarcinoma BSp73ASML contain higher levels of* miR-494* and* miR-542-3p* than the exosomes of poorly metastatic BSp73ASML CD44v4-v7 knockdown cells [75]. The mRNA and miRNA content of extracellular vesicles derived from cancer stem cells also differ from those derived from differentiated cancer cells.* miR-29a*,* miR-650*, and* miR-151* are associated with tumor invasion and metastases, and* miR-19b*,* miR-29c*, and* miR-151* are upregulated in renal carcinomas and stimulate formation of a lung premetastatic niche [76]. Exosomes derived from chronic myelogenous leukemia cells were shuttled into endothelial cells, causing modulation of their motility and adhesion. This process was associated with exosome content of miR-126, which was concentrated in chronic myelogenous leukemia cell exosomes [77]. Brain metastatic cancer cells released miRNA-181c-containing extracellular vesicles which disrupt the blood-brain barrier [78]. Exosome-mediated transfer of cancer-secreted miR-105 disrupts tight junctions to promote metastasis [79]. Exosomal transfer of miRNA-23b from the bone marrow promotes breast cancer cell dormancy in a metastatic niche [80]. miR-210, released by metastatic cancer cells, could be transported to endothelial cells and regulate cancer cell metastasis [81]. MiRNAs in extracellular vesicles also modulate tumor proliferation [82, 83], and several extracellular vesicle miRNAs have been recommended for cancer diagnosis or assessment of cancer prognosis [10, 26, 37].

### 4.2. The Role of Extracellular Vesicles in Transmitting Long Noncoding RNAs in the Cancer Microenvironment

LncRNAs are nonprotein coding transcripts longer than 200 nucleotides [84] which can participate in epigenetic transcriptional or posttranscriptional regulation. Extracellular vesicle lncRNAs have received less attention than miRNAs, but in the previously described sequence analysis of 14 size-selected sequencing libraries, lncRNAs were found to be the most abundant exosomal RNA species after miRNAs and ribosomal RNA, representing 3.36% of all mappable, countable RNAs [65].

As previously described for miRNAs, the lncRNA content of exosomes differs from that of donor cells, indicating selective secretion of lncRNAs [85]. In two cancer cell lines, HeLa and MCF-7, the difference between the exosome and donor cell content of six lncRNAs (*MALAT1*,* HOTAIR*,* lincRNA-p21*,* GAS5*,* TUG1*, and* CCND1-ncRNA*) was assessed in donor cells under DNA damage stress [86]. Whilst* MALAT1* is prevalent in donor cells, the* MALAT1* level in exosomes turns out to be relatively low; in contrast* lincRNA-p21* is enormously enriched in exosomes. Under cellular stress, the cellular content and selective loading of lncRNAs to extracellular vesicles was altered, indicating the packaging of lncRNAs in extracellular vesicles changes in response to the cancer microenvironment, potentially facilitating adaptation or opposition to the stress environment.

Extracellular vesicle content of lncRNA may reflect tumor growth, metastasis, and response to treatment. LncRNA* TUC339*, found in extracellular vesicles derived from hepatocellular carcinoma cells (HCC), has been implicated in tumor growth, adhesion and cell cycle progression [87, 88].* Linc-ROR*, another lncRNA enriched in extracellular vesicles from HCC, protects cancer cells from chemotherapy-induced apoptosis and cytotoxicity [89], and* MALAT1*, an lncRNA enriched in extracellular vesicles from cervical carcinoma and breast cancer cells [86], was associated with tumor metastasis and invasion [90, 91]. The potential for* MALAT1* to act as a blood-based biomarker for the diagnosis of nonsmall cell lung cancer is currently being evaluated [92].

## 5. Conclusions

**Figure 1:**
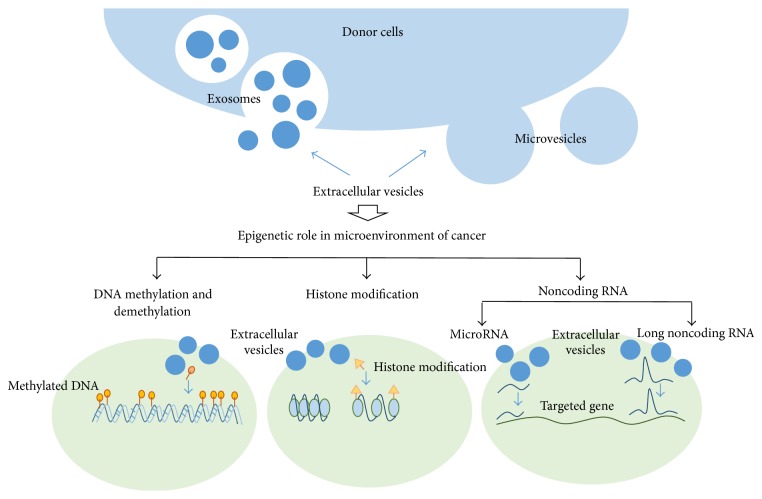
Various extracellular vesicles derived from donor cells play various epigenetic roles in microenvironment of cancer, including DNA modification, histone modification, and noncoding RNA regulations.

Extracellular vesicles originating from cancer cells contain several types of biomolecules including oncogenes and molecules capable of epigenetic reprogramming. They are shed to the cancer microenvironment and may promote cancer progression [93, 94]. Epigenetic regulation appears to play a major role in this process (Figure 1). Many of the mRNAs and proteins present in extracellular vesicles are ascribed GO biological processes related to epigenetic regulation [24]. Recipient cell methyltransferase and cytidine deaminase can be downregulated by onco-mRNA in microvesicles derived from leukemia cells [34]. LncRNA* TUC339*, found in extracellular vesicles from hepatocellular carcinoma cells, participates in tumor growth, adhesion, and cell cycle progression [87, 88]. Furthermore, detection of the biomarkers present in extracellular vesicles represents a promising, noninvasive method of cancer diagnosis. For instance, methylated* LINE1* and methylated* SOX17* DNA accumulated in gastric juice-derived exosomes [37], and* lincRNA-p21* was detected in extracellular vesicles from cervical carcinoma cells and breast cancer cells [86]. In addition, due to their capacity to mediate intercellular communication, extracellular vesicles may represent targets for therapeutic intervention, and both native extracellular vesicles and artificially engineered vesicles represent promising new tools for drug delivery [22, 23].
